# Resolvin D2 Restrains Th1 Immunity and Prevents Alveolar Bone Loss in Murine Periodontitis

**DOI:** 10.3389/fimmu.2018.00785

**Published:** 2018-04-25

**Authors:** Gabriel Mizraji, Oded Heyman, Thomas E. Van Dyke, Asaf Wilensky

**Affiliations:** ^1^Department of Periodontology, Faculty of Dental Medicine, Hebrew University of Jerusalem – Hadassah Medical Center, Jerusalem, Israel; ^2^Institute of Dental Sciences, Faculty of Dental Medicine, Hebrew University of Jerusalem – Hadassah Medical Center, Jerusalem, Israel; ^3^Forsyth Institute, Cambridge, MA, United States

**Keywords:** specialized pro-resolving lipid mediators, resolvin, periodontitis, alveolar bone loss, immune response

## Abstract

Periodontitis is an infectious inflammatory disease of the supporting structures of the teeth. Resolvins are part of a large family of specialized pro-resolving lipid mediators that enhance active resolution of inflammation and return of inflammatory lesions to homeostasis. In this paper, we demonstrate that resolvin D2 (RvD2), a product of docosahexaenoic acid (DHA) metabolism, prevents alveolar bone loss in *Porphyromonas gingivalis-*induced experimental periodontitis. Investigations of the immune mechanism of RvD2 actions reveal that 6 weeks after infection, the gingiva of RvD2-treated mice exhibit decreased CD4^+^ T-cells as well as lower RANKL expression levels and higher osteoprotegerin expression levels. Systemically, RvD2 prevents chronic secretion of IFN-γ and rapidly restores IFN-α levels, without dampening the *P. gingivalis*-specific immune response. In the gingiva, immediately after *P. gingivalis* inoculation, RvD2 regulates the mRNA expression of IFN-γ, IL-1β, TNF-α, and IL-10, hence contributing to maintaining local homeostasis. Moreover, RvD2 treatment reduces local neutrophil numbers, whereas pro-resolving macrophage counts were increased. These findings suggest that RvD2 resolves innate inflammatory responses, inhibiting systemic and gingival Th1-type adaptive responses that are known to mediate alveolar bone loss in this model.

## Introduction

Periodontitis is a chronic, polymicrobial infectious-inflammatory disease of the supporting structures of the teeth (1). *Porphyromonas gingivalis* (*P. gingivalis)*, a Gram-negative anaerobic bacteria, is most closely associated with chronic periodontitis and considered to be a keystone pathogen (2). As such, it is thought to initiate microbial dysbiosis leading to a destructive chronic inflammatory lesion and bone loss (2). Although periodontitis is one of the most common chronic diseases in humans (3), the pathogenesis of the disease is not fully understood. Recently, cumulative evidence suggests that interferons (IFNs) have an important role in the course of periodontitis. The concentrations of IFN-γ are significantly higher in serum samples and gingival tissue biopsies obtained from periodontitis patients compared to people without periodontitis (4). Moreover, high levels of IFN-α have been reported in the gingiva of people with periodontitis compared to people without periodontitis (5), as well as in the plasma (6, 7). Peripheral blood neutrophils obtained from people with active periodontitis have an hyperactive phenotype that is consistent with the IFN-α signature in their blood (6). More recently, we have shown in a murine model of *P. gingivalis*-induced experimental periodontitis that diseased mice produce high levels of IFN-γ and highly express type-1 IFNs for a prolonged period of time, compared to uninfected mice (5). The elevated expression of type-1 IFNs dysregulates several gingival immunological functions, induces prolonged priming of CD4^+^ Th1 cells by DCs, increases basal levels of IFN-γ, and elevates receptor activator of NF-κB ligand (RANKL) expression (5). Recently, it has been suggested that expression of RANKL by CD4^+^ T-cells can directly modulate the tightly regulated network of bone homeostasis (8, 9), and that osteoprotegerin (OPG) is a key regulator of the differentiation, activation, and survival of osteoclasts and their precursors. *P. gingivalis* was reported to modulate the RANKL-OPG axis during experimental periodontitis and affect bone loss (10–12).

Resolvins are the products of the metabolism of omega-3 (ω-3) polyunsaturated fatty acids in the diet that are produced later in the inflammatory response and dominate eicosanoid mediators in resolving inflammatory exudates (13). Resolvins are part of a large family of specialized pro-resolving lipid mediators (SPM); they provide the negative feedback loop inducing the active resolution of the acute inflammatory response (14), initiating the cessation of leukocyte infiltration and recruitment of monocytes (15, 16). These natural mediators of resolution of inflammation actively promote tissue repair and bacterial clearance and enhance, rather than inhibit, host defense (17). In addition, several SPMs including resolvins can also impact the responses of adaptive immune cells. RvD2 (RvD1 and MaR1) reduced TNF-α and IFN-γ production by human CD4^+^ and CD8^+^ T-cells upon stimulation with PMA/ionomycin (18). Moreover D-series resolvins were found to play a pivotal role in cell differentiation and promote resolution by preventing the generation of activated Th1 and Th17 cells and enhancing the differentiation of regulatory T-cells (18). Resolvins of the D-series are derived from docosahexaenoic acid (DHA) and include RvD2 (7S,16R,17S-trihydroxy-4Z,8E,10Z,12E,14E,19Z-DHA), which binds to the cell surface receptor GPR18/DRV2 providing significant protection in infection-induced inflammation (19). Small animal models have shown that control of inflammation with specialized pro-resolving lipid mediators prevented and treated experimental periodontitis (20–23). However, characterization of the actions of RvD2 treatment on the immune response during *P. gingivalis* gavage-induced experimental periodontitis was never tested. We specifically focus on RvD2 because of its potency, its effectiveness in reducing Th1/Th17 polarization (18), and previously reported actions on IFNs in sepsis, burn wounds, and progression of atherosclerosis (19, 24–26).

This study aims to characterize the pathways of alveolar bone loss, the RANKL/OPG axis, and the regulation of cytokines and T-cells in the inflammatory lesion that are modified by RvD2.

## Materials and Methods

### Mice

Seven to eight weeks old female BALB/c or B6 mice were purchased (Harlan, Israel) and were maintained under specific pathogen-free (SPF) conditions. The T-cell receptor transgenic mouse strains OT-I and OT-II were bred in-house and maintained under SPF conditions. All animal protocols were approved by the Institutional Animal Care and Ethics Committee.

### Antibodies and Reagents

The following fluorochrome-conjugated anti-mouse monoclonal antibodies for flow cytometry were purchased from BioLegend (USA): CD45.2 (104), CD4 (GK1.5) I-A^d^ (39-10-8), Ly6G (1A8), Ly6C (HK1.4), Gr-1 (RB6-8C5), CD3 (17A2), CD11b (M1/70), CD8α (53-6.7), IFN-γ (XMG1.2), FoxP3 (MF-14), F4/80 (BM8). For immunoblotting, the following primary antibodies were purchased from Santa Cruz Biotechnology: polyclonal goat anti-mouse IRF4 (H-140), IRF5 (H-56), and rabbit polyclonal anti-actin (I-19). Secondary antibodies were purchased from Abcam.

### Experimental Periodontitis

**Figure 1:**
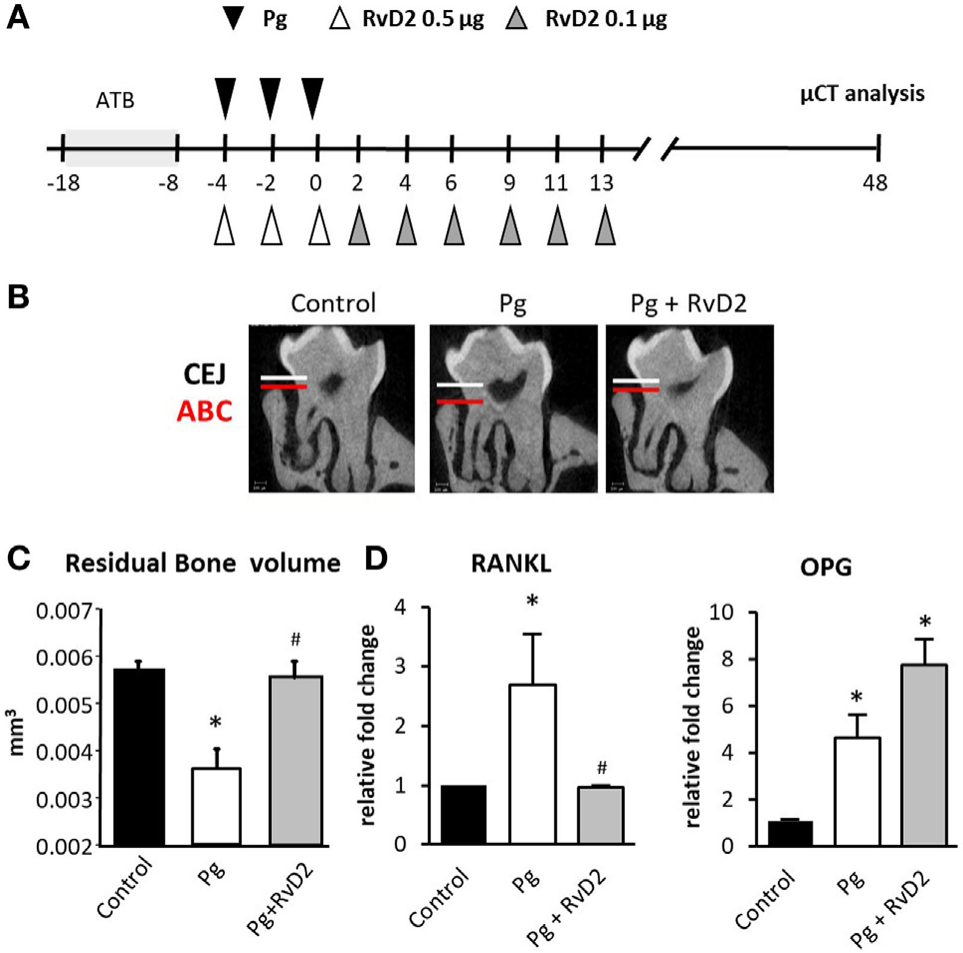
Resolvin D2 prevents alveolar bone loss by shifting the RANKL/osteoprotegerin (OPG) ratio. **(A)** Schematic presentation demonstrating the experimental system. Briefly, mice were treated with antibiotic (ATB) in the drinking water *ad libitum* for 10 days, followed by 3 days of antibiotic-free period. The mice were then infected *via* oral gavage with 10^9^ colony-forming units of *Porphyromonas gingivalis* in 2% carboxymethylcellulose either with or without RvD2 0.5 µg (i.p.) for the first week and three injections per week with 0.1 µg of RvD2 (i.p.) during the following 2 weeks. Control mice received vehicle only. **(B)** Representative μCT sections of the second upper molar demonstrating the impact of RvD2 treatment on the distance between the cemento-enamel junction and alveolar bone crest. **(C)** 3D quantification of the residual alveolar bone volume 6 weeks after infection. Significant lower residual bone volume was measured in the *P. gingivalis-*infected group compared to the other groups. Data are presented as the residual volume of alveolar bone in the buccal plate and represent the means of eight mice per group ± SEM. Results are representative of three independent experiments, *p < 0.05 relative to control group. ^#^*p* < 0.05 relative to Pg group. **(D)** RANKL and OPG mRNA expression was quantified by Real-Time *q*PCR 6 weeks after infection (gene/TBP) in the jaw soft and hard tissues surrounding the molars. The presented data represent the means of 6 mice per group ± SEM. Results are representative of two independent experiments, *p < 0.05.

Experimental periodontitis was induced as described previously (27). In brief, mice were treated with trimethoprim (0.16 mg/mL) and sulfamethoxazole (0.8 mg/mL) solution (Resprim; Teva Pharmaceutical Industries) in the drinking water for 10 days, followed by 3 days without antibiotics. 1 × 10^9^ colony-forming units of *P. gingivalis* 53977 were introduced *via* oral gavage, three times at 2-day intervals in 200 µL of 2% (wt/vol) carboxymethylcellulose (CMC) solution (Sigma). Control mice were treated with CMC 2% vehicle alone. The Pg + RvD2 group was treated with three doses of 0.5 µg RvD2 (Cayman Chemical) in a total volume of 150 µl of sterile saline solution intraperitoneally (i.p.) followed by six intraperitoneal doses of 0.1 µg of RvD2 over the next 2 weeks (Figure 1A). The Pg group received 150 µl of sterile saline solution i.p. in the same regimen as the Pg + RvD2. Control mice received 3 oral gavages of 2% CMC 2 days a part. 6 weeks later, mice were euthanized, and the hemi-maxillae were harvested and scanned using micro-computed tomography (μCT) (μCT 40, Scanco Medical, Switzerland) for alveolar bone loss quantification.

### Quantification of Alveolar Bone Loss

For quantitative 3-dimensional analysis of the alveolar bone loss, the hemi-maxillae were examined by a desktop μCT system. The sagittal plane of the specimens was set parallel to the X-ray beam axis. The specimens were scanned at a resolution of 12 µm in all three spatial dimensions. The scans were Gaussian-filtered and segmented using a multi-level global thresholding procedure for the segmentation of enamel, dentin, and bone. Residual supportive bone volume was determined separately for either root (bucco-mesial and bucco-distal) using a direct 3-dimensional approach (28). The measured mesio-distal length of the alveolar bone was 204 and 120 µm for the mesio-buccal and the disto-buccal roots, respectively. The apical basis of the measured volume was set mesio-distally parallel to the cemento-enamel junction and bucco-palatally parallel to the occlusal plane. The results represented the residual bone above the reference plane in cubic millimeter (29).

### Isolation and Processing of Gingival Tissue

The gingivae were excised, processed for single cell suspension, and stained with antibodies, as previously described (30). Briefly, the gingivae were excised, minced, and treated with collagenase type II (2 mg/mL; Worthington Biochemicals) and DNase I (1 mg/mL; Roche) solution in PBS + 2% fetal calf serum (FCS) for 20 min at 37°C in a shaker bath. A total of 20 µL of 0.5 M EDTA per 2 mL sample was added to the digested tissues and incubated for additional 10 min. The cells were washed, filtered with a 70 µM filter, and stained with antibodies as indicated in each experiment. Foxp3 staining was performed using the Foxp3 Fix/Perm Buffer Set (BioLegend) according to the manufacturer’s instructions. Stained cells were analyzed using a BD LSR II flow cytometer. Fetal calf serum (FCS) 3.0 files were analyzed using FlowJo vX (Treestar).

### Macrophage Isolation

After gingival processing as described previously, macrophages were isolated using a BD FACS Aria II cell sorter. After exclusion of dead cells, cells expressing CD45^+^, F4/80^+^, and I-A^d+^ were sorted.

### Western Blot

Gingival macrophages were isolated as described previously and homogenized in ice-cold lysis buffer (50 mM Tris pH 7.5, 150 mM NaCl, 1% Triton X-100, 0.5% NP-40, 0.1% SDS, 0.5 mM EDTA) supplemented with a protease inhibitor cocktail (Sigma). Following lysate incubation for 30 min on ice, tissue debris was removed by centrifugation (15,000 *g*, 30 min, 4°C) and protein concentration in the supernatant was determined using Pierce BSA Protein Assay Kit (Thermo Scientific). Samples of 30 µg of total protein were loaded onto 4–20% acrylamide gel (Geba) and subjected to SDS-PAGE. Proteins were transferred to a PVDF membrane (Millipore), the membrane was blocked in 5% skim milk (BD) for 30 min at room temperature (RT), and reacted with primary antibody overnight at 4°C. The membrane was then washed three times in tris-buffered saline-Tween 20 (TBST) for 15 min at RT, incubated with secondary HRP-conjugated antibody in blocking buffer for 1 h at RT and washed three times in TBST before the blots were reacted with enhanced chemiluminescence substrate (Western blot detection kit, Advansta). Images were captured using a ChemiDoc™ MP Image System (Bio-Rad). Relative density was calculated using ImageJ software.

### RNA Extraction and Real-Time *q*PCR (RT-*q*PCR)

For RNA isolation from the maxilla, soft or both soft and hard tissues were homogenized in 1 mL TRI reagent (Sigma) using electric homogenizer, and RNA was extracted according to the manufacturer’s instructions. For RNA isolation from lymph nodes, tissue was homogenized manually in 0.5 mL of TRI reagent (Sigma). cDNA synthesis was performed using the qScript™ cDNA Synthesis Kit (Quanta-BioSciences Inc™). RT-*q*PCR reactions (20 µl volume) were performed using Power SYBR Green PCR Master Mix (Quanta-BioSciences IncTM.). The following reaction conditions were used: 10 min at 95°C, 40 cycles of 15 s at 95°C, and 60 s at 60°C. The gingival samples were normalized to TBP (TATA box binding protein) gene as control and the lymph node samples to β-actin (Figure S1 in Supplementary Material). The 2^−ΔΔCT^ method was used to calculate relative gene expression.

### Serum Analysis

Six weeks after infection, blood was drawn from mice and the sera were stored at −80°C. Ninety-six-well plates (Nunc) were coated overnight at 4°C with 1 µg of *P. gingivalis* 53977 lysate/well in 0.1 M bicarbonate buffer (pH 9). The plates were washed twice with PBST and blocked with PBS 10% FCS (2 h at room temperature). Subsequently, mouse serum samples diluted serially in PBS 10% FCS were added to the wells for 3 h incubation at RT. This was followed by four washes in PBST and the addition of anti-mouse peroxidases-conjugated IgG antibody (Jackson ImmunoResearch). After incubation for 2 h at RT, the plates were washed five times and 100 µL/well of tetramethyl benzidine (TMB) solution (Southern Biotech) was added for 5 min, followed by the addition of 100 µL of TMB stop solution (Southern Biotech). Absorption was read at 450 nm using an iMARK microplate reader (Bio-Rad).

### *Ex Vivo* Antigen Presentation Assay

Cervical lymph nodes were collected from the mice and treated with collagenase type II (1 mg/mL) and DNase I (1 mg/mL) solution in PBS + 2% FCS for 20 min at 37°C in a shaker bath. 20 µl of 0.5 M EDTA per 2 mL sample was added to the digested lymph nodes and incubated for an additional 10 min. The cells were then washed and filtered. CD11c^+^ cells were enriched from the digested lymph nodes by positive isolation using MACS MicroBeads according to the manufacturer’s instructions (Miltenyi Biotec, Germany). CD4^+^ T-cells were purified from naive mice by negative selection with the EasySep mouse CD4^+^ T-cell enrichment kit, according to the manufacturer’s instructions (StemCell Technologies, Canada). The purified T-cells (1 × 10^5^/well) were incubated with the various DC subsets with the enriched CD11c^+^ cells (1 × 10^4^/well) in 96-well U-bottom plates (Nunc, Thermo fisher). The cultures were then incubated at 37°C, 5% CO_2_ for 60 h, the supernatants were collected and stored at −80°C immediately.

### Cytokine Secretion by Cultured Splenocytes

Splenocytes cells were collected from infected mice 6 weeks after inoculation. The cells were cultured for 60 h at 37°C, 5%CO_2_ with complete medium either in the presence or absence of the *P. gingivalis* antigen RgpB. The supernatants were collected and stored at −80°C immediately, for further analysis.

### Protein Quantification by ELISA

The level of IFN-γ in the supernatants of the *ex vivo* antigen presentation assay and the splenocyte re-stimulation assay was measured using an ELISA MAX mouse IFN-γ kit (BioLegend) according to the manufacturer’s instructions. Cytokine levels were determined using standard curves of recombinant cytokines and are expressed as picogram per milliliter.

### Statistical Analysis

Data were expressed as mean ± SEM. Statistical tests were performed using one-way analysis of variance with the Student–Newman–Keuls method for correction of multiple testing or Student *t*-test as indicated. A *p*-value < 0.05 was considered significant.

## Results

### RvD2 Shifts RANKL/OPG Ratio and Prevents Alveolar Bone Loss in Experimental Periodontitis

In order to examine the ability of RvD2 to modulate immune responses during experimental periodontitis, we infected mice with *P. gingivalis via* oral gavage and administrated RvD2 intraperitoneally during and after gavage as described in Figure 1A. Treatment with RvD2 prevented *P. gingivalis*-induced alveolar bone loss (Figures 1B,C). Next, we measured RANKL and OPG expression in the gingiva. Our results show that RvD2 treatment reduced RANKL expression significantly, while OPG levels were upregulated (Figure 1D). These results suggest that RvD2 modulates the RANKL/OPG axis, and prevents further alveolar bone loss.

### RvD2 Decreases T-Cell Activation, Reduces Systemic CD4^+^ T-Cell Responses and Inhibits Gingival Inflammation

**Figure 2:**
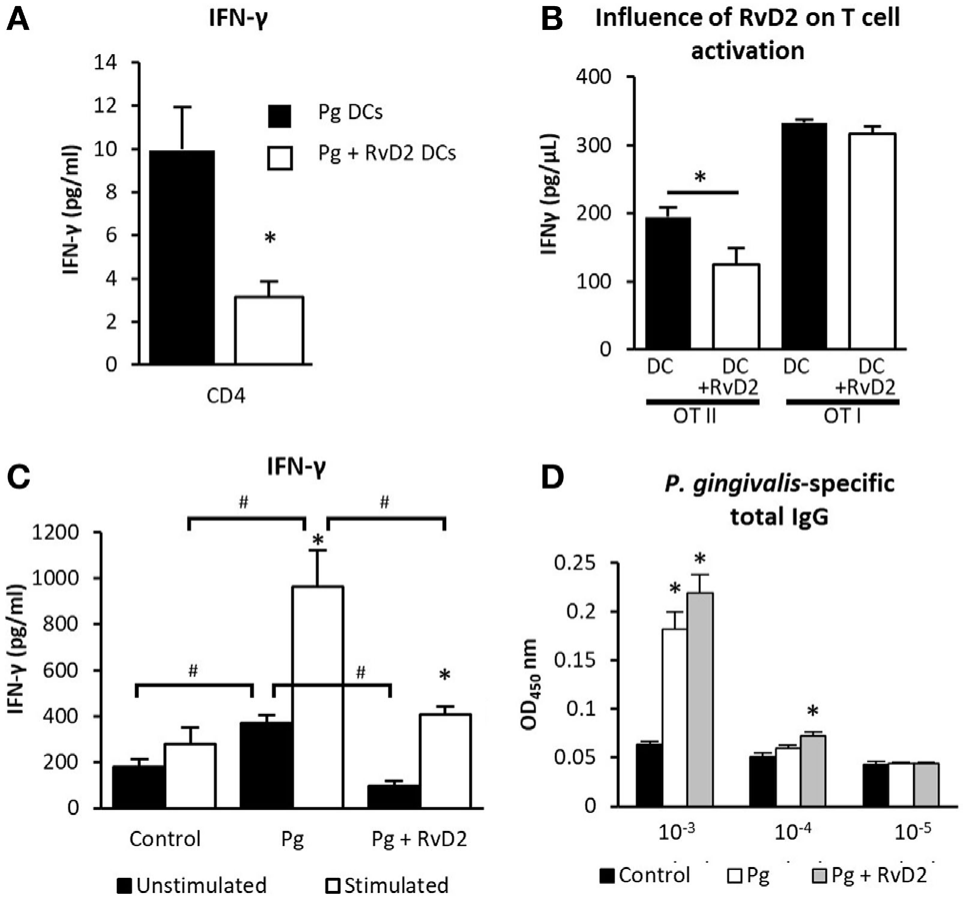
Resolvin D2 inhibits T-cell IFN-γ spontaneous secretion. **(A)** 4 days after the last exposure, CD11c^+^ APCs from cervical lymph nodes from Pg or Pg + RvD2 groups, were isolated and challenged with CD4^+^ T-cells harvested from the lymph nodes of naive mice, then those cells were incubated for 72 h, at 37°C, 5% CO_2_ and IFN-γ was measured in the supernatant. Data represent the mean ± SEM of two independent experiments. **(B)** APCs were enriched from the lymph nodes of B6 mice and challenged with CD4^+^ cells enriched from OT II or CD8^+^ cells enriched from OT I mice, and then cultured together in the presence of its specific antigen with or without RvD2 and incubated for 72 h, at 37°C, 5% CO_2_. The levels of IFN-γ in the supernatants were quantified by ELISA. Data represent the mean ± SEM of two independent experiments. **(C)** Six weeks after infection, splenocytes were harvested and incubated (2 × 10^6^ cells per well) with or without RgpB antigen for 72 h, the levels of IFN-γ in the supernatants were quantified by ELISA. The presented data represent the means of six mice per group ± SEM. Results are representative of two independent experiments. **(D)** Serum was collected 6 weeks after infection and total *Porphyromonas gingivalis-*specific IgG was tittered with ELISA. The presented data represent the means of six mice per group ± SEM. Results are representative of two independent experiments, *p < 0.05 ^#^*p* < 0.05.

Since *P. gingivalis*-induced alveolar bone loss is known to be mediated by adaptive immune responses and in particular CD4^+^ T-cells (31–33), we first examined the ability of CD11c^+^ dendritic cells (DCs) purified from the cervical lymph nodes of *P. gingivalis-*infected mice to prime naive CD4^+^ T-cells (without adding specific antigens) either treated or not. The analysis of secreted IFN-γ revealed that RvD2 exposed DCs were less effective in priming CD4^+^ T-cells (Figure 2A). The reduced priming prevents Th1 chronic activation and consequently its deleterious effect, as was previously described (5). To better understand the specific actions of RvD2 on T-cell priming by DCs and to further examine whether RvD2 hampers their ability to present antigens, we isolated DCs from lymph nodes of B6 mice, co-cultured them with either OVA-specific OT-I CD8^+^ T-cells or OVA-specific OT-II CD4^+^ T-cells in the presence of ovalbumin peptides and RvD2. IFN-γ production by CD4^+^ T-cells was significantly reduced in the presence of RvD2, whereas no impact was found on CD8^+^ T-cells (Figure 2B). Next, splenocytes were harvested from mice 6 weeks after infection and re-stimulated *ex vivo* with *P. gingivalis* RgpB antigen to assess T-cell responses. Splenocytes isolated from *P. gingivalis* infected mice secreted high levels of IFN-γ upon re-stimulation, while RvD2 treatment significantly reduced IFN-γ secretion (Figure 2C). Furthermore, RvD2 treatment diminished spontaneous IFN-γ secretion by splenocytes. Collectively, these results indicate that resolvin has a direct impact on T-cell priming by DCs, thus inhibiting the chronic production of IFN-γ. Finally, since T helper cells are important for antibody class switching, we evaluated the actions of RvD2 on *P. gingivalis*-specific IgG titers in infected mice. As depicted in Figure 2D, RvD2 did not interfere with antibody production against *P. gingivalis*. These findings suggest that RvD2 prevents alveolar bone loss by modulating Th1 responses without interfering with the Th2 immune responses.

**Figure 3:**
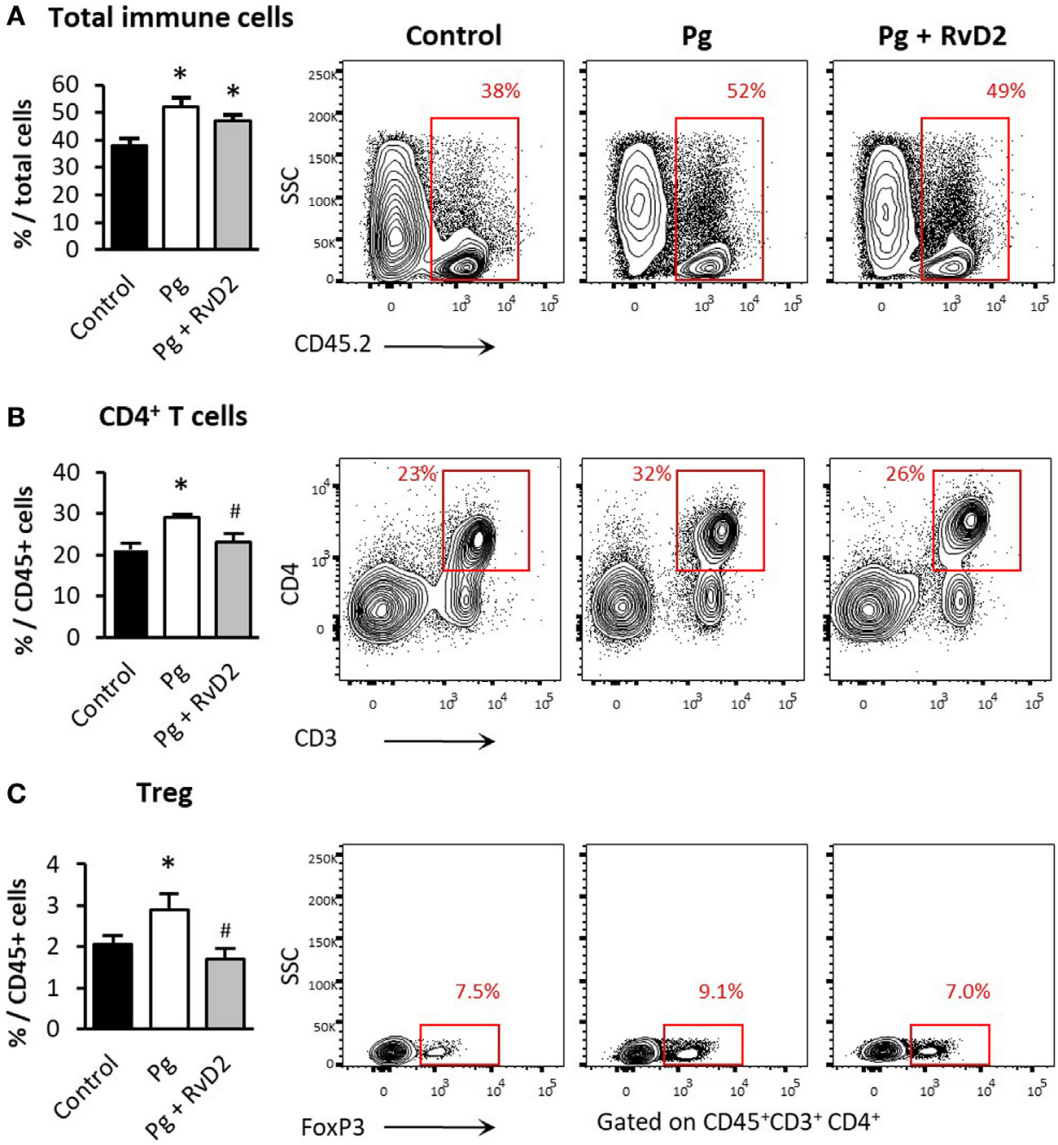
Resolvin D2 treatment prevents CD4^+^ and Treg accumulation in the gingiva. Quantification by flow cytometry analysis of **(A)** total immune cells, **(B)** CD4^+^ T-cells, and **(C)** Treg cells, in line with their representative FACS plots 6 weeks after the last infection (Treg FACS plots are gated on CD45^+^CD3^+^CD4^+^ cells). Data represent the mean of six mice per group ± SEM of two independent experiments, *p < 0.05 relative to control group. ^#^*p* < 0.05 relative to Pg group.

Next, we analyzed the local immune response in the gingiva 6 weeks after infection. While RvD2 treatment did not prevent the typical increase of immune cells in the gingiva (Figure 3A), it had considerable impact on the type of T-cell accumulating in the tissue. Specifically, gingiva of RvD2-treated mice contained less CD4^+^ T-cells (Figure 3B) and Foxp3^+^CD4^+^ Treg cells, which are known to accumulate in the gingiva during inflammation and experimental periodontitis (Figure 3C) (5, 34, 35).

### RvD2 Treatment Prevents Neutrophil Accumulation in the Gingiva and Promotes M2 Macrophage Accumulation

**Figure 4:**
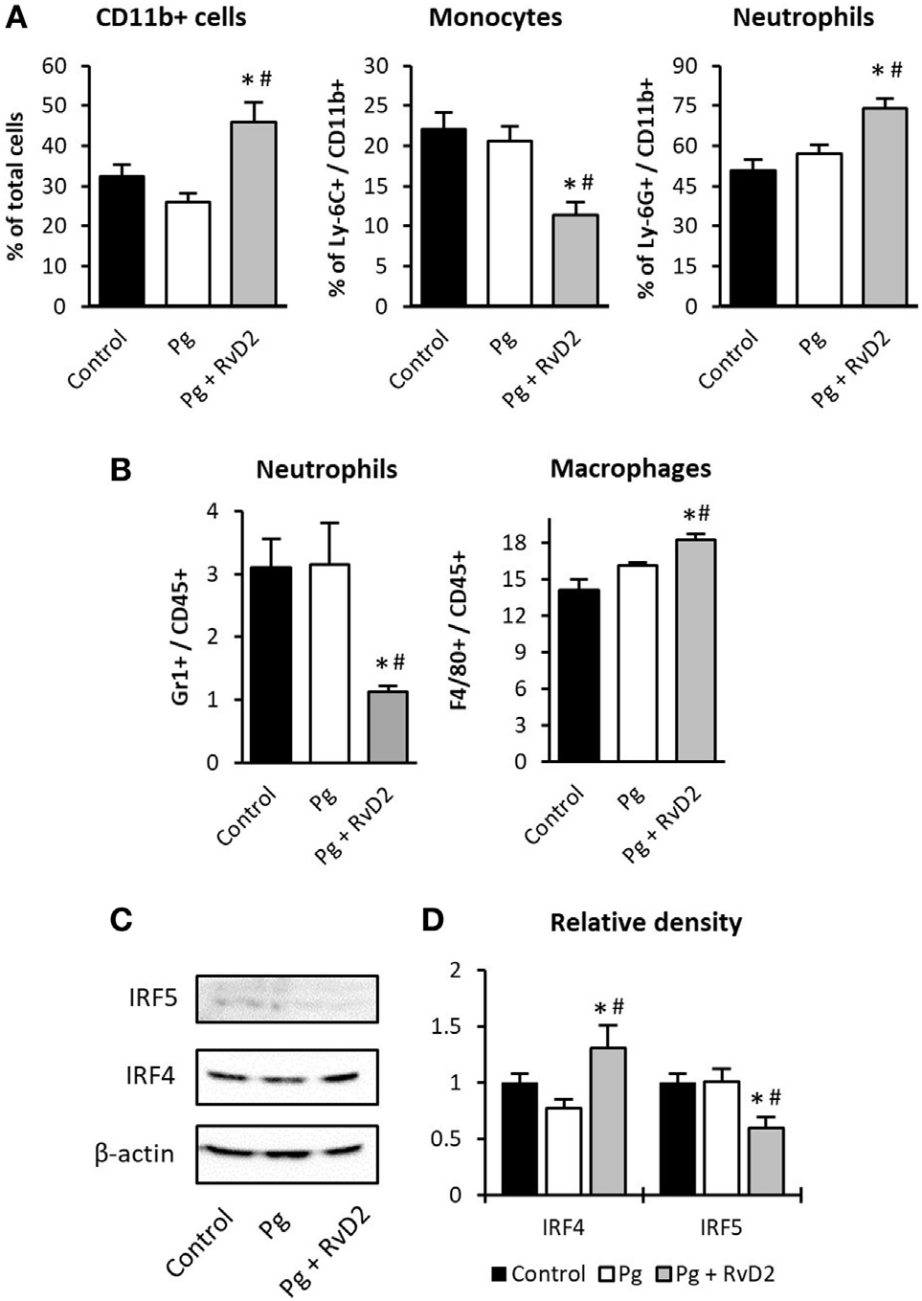
Resolvin D2 dampens neutrophil recruitment to the gingiva. Three days after the last exposure, **(A)** blood cells and **(B)** gingival tissues were analyzed by flow cytometry. Data represent the mean of five mice per group ± SEM of three independent experiments. **(C)** Gingival macrophages were FACS sorted (CD45^+^, F4/80^+^, I-A^d+^), and processed with lysis buffer for protein extraction, after protein quantification, 30 µg per sample were immunoblotted against IRF4, IRF5, and β-actin. The blot is representative of three pooled samples of two independent experiments. **(D)** IRF4 and IRF5 protein relative density (gene/actin) of gingival isolated macrophages, *p < 0.05 relative to control group. ^#^*p* < 0.05 relative to Pg group.

As resolvins are involved in active resolution of inflammation, the timing of their production is critical for effective and protective immunity. In light of this, we sought to determine the impact of RvD2 treatment on early immunological events in the gingiva and blood. Three days after infection, RvD2 treatment increased the frequency of myeloid CD11b^+^ cells in the blood (Figure 4A). The elevation was attributable to an increase in circulating neutrophils rather than monocytes. Nevertheless, despite the increase in neutrophils in the blood, the percentage of neutrophils in the gingiva was significantly lower in RvD2-treated mice compared to control group (Figure 4B). The number of macrophages in the gingiva increased after RvD2 treatment (Figure 4B). Further analysis revealed that the macrophages have elevated protein levels of transcription factor IRF4, a key adaptor in M2 polarization (36), whereas the levels of IRF5, a key adaptor in M1 polarization (37), were decreased (Figures 4C,D). The elevated M2 macrophages in the gingiva are consistent with the reduced neutrophils in the tissue, since M2 macrophages exhibit enhanced ability to engulf bacteria and apoptotic neutrophils (38).

### RvD2 Contributes to Homeostasis by Regulating the Expression of Pro and Anti-Inflammatory Cytokines

**Figure 5:**
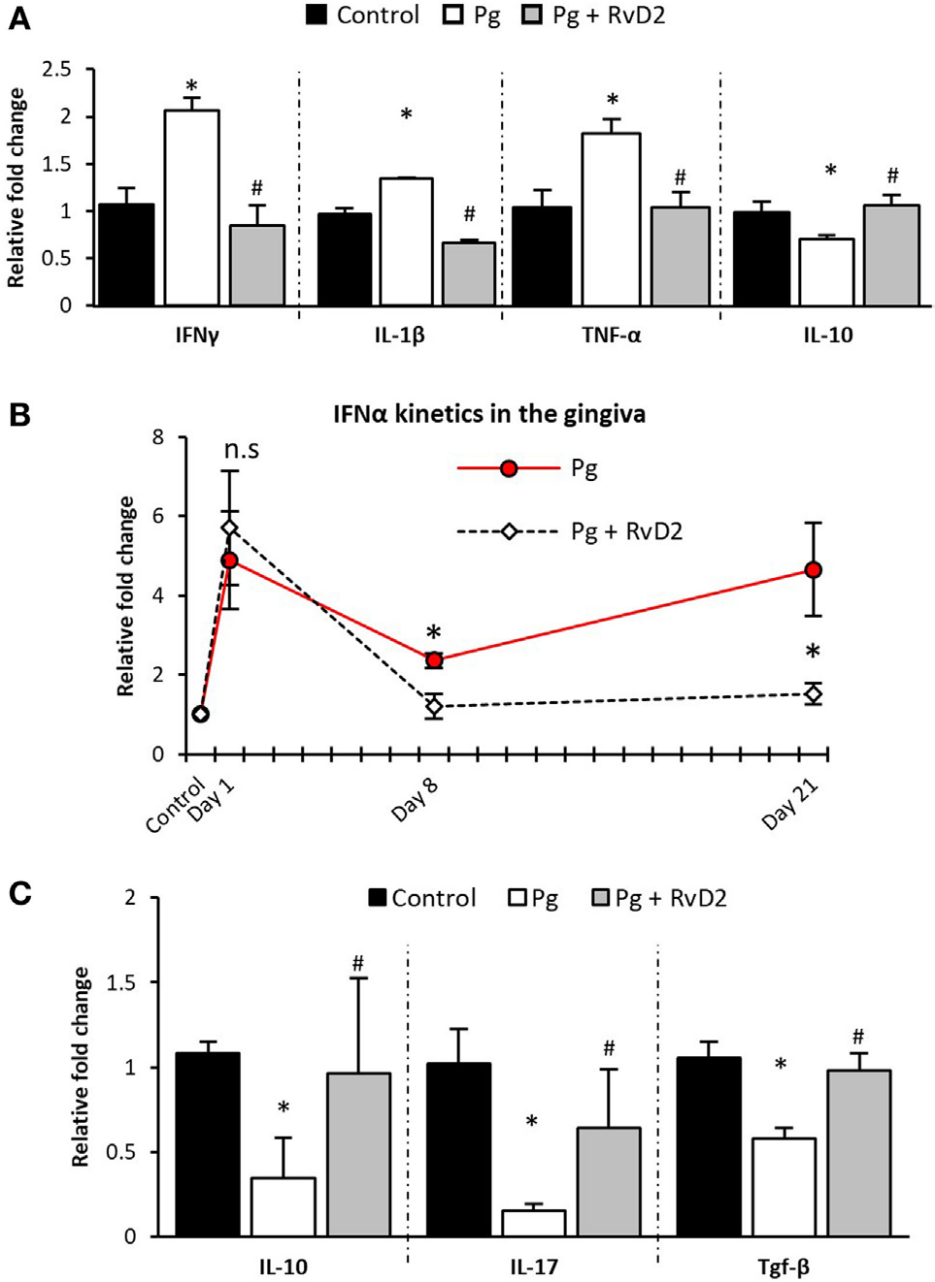
Resolvin D2 promotes resolution of the inflammatory environment in the gingiva and in the lymph nodes by regulating the expression of pro and anti-inflammatory cytokines. Four days after the last exposure, soft gingival tissues and lymph nodes were processed for RNA extraction, **(A)** gingival IFN-γ, IL-1β, TNF-α, and IL-10 mRNA expression levels were quantified and presented using real-time *q*PCR analysis (gene/TBP). **(B)** Analysis of the kinetics of IFN-α mRNA levels in the gingival soft tissue. **(C)** Expression of IL-10, IL-17, and TGF-β (gene/β-actin) in the lymph nodes. The presented data represent the means of five mice per group ± SEM. Results are representative of two independent experiments, *p < 0.05 relative to control group. ^#^*p* < 0.05 relative to Pg group.

T and B-cells and their cytokines are reported to be involved both directly and indirectly in the inflammation-induced bone resorption seen in periodontal disease (39–41). In order to evaluate the impact of RvD2 treatment on the cytokine profile, we examined the expression of pro and anti-inflammatory cytokines in the gingiva 4 days following *P. gingivalis* infection. RvD2 administration prevented increased expression of the pro-inflammatory cytokines, IFN-γ, IL-1β, and TNF-α, and the decrease of the anti-inflammatory cytokine IL-10 (Figure 5A). Interestingly, RvD2 receptor GPR18 expression is upregulated immediately after repetitive challenges with *P. gingivalis* (Figure S2 in Supplementary Material), as was previously demonstrated under inflammatory conditions (42) conferring sensitization to its biological agonist. Four days after infection there are no differences in GPR18 expression. GPR18 expression remains unchained after sustained RvD2 administration, in line with previous results in rat hearts using GPR18 agonist (43). Most of the G protein-coupled receptors including GPR18 undergo downregulation when chronically exposed to their agonist (44).

Furthermore, RvD2 treatment rapidly restored IFN-α level in the gingiva, in contrast to *P. gingivalis* challenge that raised gingival IFN-α levels for at least 3 weeks (Figure 5B). In the lymph nodes, RvD2 treatment preserved the expression of key immune molecules, such as IL-10, IL-17, and TGF-β (Figure 5C). Taken together, treatment with RvD2 prevented the pro-inflammatory shift in cytokine expression following *P. gingivalis* infection and maintained tissue homeostasis.

## Discussion

In this paper, we demonstrate that the D-series resolvin, RvD2, is protective against *P. gingivalis* induced periodontal bone loss in the mouse. RvD2 reduces the RANKL/OPG ratio in gingival tissues favoring bone preservation and RvD2 downregulates IFN-γ secretion by CD4^+^ T-cells. Neutrophils in circulation are increased by RvD2 administration, but neutrophils in tissues are markedly decreased. The opposite is observed for macrophages, where RvD2 decreases monocytes in circulation and increases M2 macrophages, in gingiva. mRNA levels of IFN-γ, IL-1β, and TNF-α are decreased in gingiva after RvD2 treatment and IL-10 is increased. The IFN-α response in gingiva is normalized at 8 days in RvD2 treated mice, while it is still elevated at 21 days in untreated periodontitis. These findings suggest that RvD2 restrains excessive innate inflammatory responses, inhibiting systemic and gingival Th1-type adaptive responses that are known to mediate alveolar bone loss in this model without inhibiting protective immunity.

Periodontitis is an infectious inflammatory disease that leads to inflammatory bone loss around teeth. The early gingivitis lesion is a neutrophil dominated lesion with soft tissue breakdown and significant loss of collagen due to neutrophil collagenase and elastase secretion (45). Although the trigger for transition from gingivitis to periodontitis has not been elucidated, it is known that it is characterized by a continuous and uncontrolled response by neutrophils leading to “neutrophil-mediated tissue injury” (46, 47). Since neutrophils are key players in the pathogenesis of periodontal disease, we tested the impact of treatment with RvD2 on neutrophils. Our results show that RvD2 administration increases circulating neutrophil numbers, but they do not migrate and accumulate the gingiva (Figures 4A,B). This finding is consistent with mobilization of neutrophils from the bone marrow with accumulation of neutrophils in the circulation, but reduced capacity to infiltrate the tissue, consistent with observations suggesting that resolvins prevent endothelial transmigration (48, 49) and may represent a possible mechanism by which RvD2 prevents *P. gingivalis* induced alveolar bone loss. Further analysis revealed that the elevated macrophages in the tissue have increased IRF4/IRF5 protein ratio (Figures 4C,D) indicating a phenotypic shift to M2 polarization, acting to resolve the inflammation (50). These findings are in agreement with previous work demonstrating the ability of RvD2 and other specialized pro-resolving lipid mediators to polarize macrophages to M2-like cells, to further increase monocyte recruitment and enhance macrophage phagocytosis and efferocytosis (51, 52).

The efficient clearance of neutrophils from the gingiva is essential for the return of tissues to homeostasis and healing. The rapid termination of the local innate immune response and the ability of RvD2 to impair DC maturation *via* down-modulation of MHC class II expression, as was previously shown with RvD1 (53), could explain the reduced antigen presentation and CD4^+^ T-cell priming by DCs as demonstrated in Figures 2A,B. These findings are further supported by our *ex vivo* data in *P. gingivalis* infected mice; 6 weeks after infection, RvD2 treatment reduces IFN-γ secretion by both stimulated and unstimulated splenocytes (Figure 2C). These results are in line with a recent report showing that RvD2 prevents Th1 cell differentiation and activation (18). Collectively, our data suggests that RvD2 limits the observed excessive innate immune response and further pathological activation of the Th1 response due to the repeated exposure to *P. gingivalis* during consecutive oral gavages.

In cases where the innate immune response is insufficient to eliminate the infection, chronic inflammation ensues with an active adaptive immune maintenance of inflammation characterized by B-cells, T-cells, and secretion of pro-inflammatory cytokines (39–41). Our results are consistent with these observations and demonstrate that treatment with RvD2 reduces the frequency of CD4^+^ T-cells and Foxp3^+^CD4^+^ Treg cells in the gingiva 6 weeks after infection (Figures 3B,C). In contrast with our results, RvD2 has been shown to promote Treg differentiation (18). However, we believe that this difference is based on the timing of the analysis. We have found elevated number of Tregs in the gingiva 2–3 weeks after *P. gingivalis* infection and RvD2 treatment (data not shown), these counts seem to normalize later on reaching homeostatic levels after 6 weeks. Since Treg cells downregulate inflammation, their reduction in the RvD2-treated group suggests a normal and homeostatic gingival immune cell content. Examination of the gingivae for pro and anti-inflammatory cytokine expression 4 days post infection revealed that treatment with RvD2 maintained tissue homeostasis by preventing increased expression of the pro-inflammatory cytokines, such as IFN-γ, IL-1β, and TNF-α and decreased expression of IL-10 (Figure 5A).

Recently, we have shown that type-1 IFNs play a major role in the pathogenesis of periodontal disease by disrupting innate immunological functions, constitutively priming CD4^+^ T-cells by DCs leading to elevated RANKL expression and, subsequently, alveolar bone loss (5). Blocking type-1 IFN signaling prevented the destructive Th1 immune response and alveolar bone loss (5). Interestingly, the active resolution of inflammation induced by RvD2 has the capacity to restore the homeostatic levels of IFN-α after repetitive *P. gingivalis* challenges, and thus prevents the chronic production of IFN-γ and disease progression. Type-1 IFNs are important to the constitutive priming of CD4^+^ T-cells by DCs, since high levels of type-1 IFNs promote DCs maturation (54) and consequently contribute to the ability of these cells to present antigens and prime T-cells. The fact that treatment with RvD2 reduces the expression of IFN-α immediately after infection is in agreement with our previous results showing reduced antigen presentation by DCs after RvD2 treatment (Figures 3A,B). Moreover, we found restored levels of IL-10 in the lymph nodes 4 days after infection in RvD2-treated mice. This cytokine is known to be a negative regulator of IFN-α (55) preventing re-saturation of type-1 IFN levels. We suggest that the well-regulated kinetics of IFN-α enables a protective inflammatory response without the detrimental effects of unrestrained IFN-α production seen in periodontitis. The mechanism by which RvD2 influences the production of IFN-α is still unknown. However, we show that RvD2 treatment has an impact on IFN-α level, and thus can prevent excessive activation of the Th1 response.

With regards to bone loss, it has been suggested that expression of RANKL on CD4^+^ T-cells can directly modulate the tightly regulated network of bone homeostasis (8, 9, 56), and that osteoprotegerin (OPG) is a key regulator of the differentiation, activation, and survival of osteoclasts and their precursors. *P. gingivalis* was reported to modulate the RANKL-OPG axis during experimental periodontitis and affect bone loss (11, 12, 57). In this study, we show that treatment with RvD2 decreased the RANKL/OPG ratio. These results suggest that RvD2 treatment prevents osteoblast-mediated and T-cell-mediated signaling of osteoclast formation by RANKL leading to alveolar bone loss.

Taken together, the data suggest that treatment with RvD2 prevents destructive immunity and alveolar bone loss in experimental periodontitis in mice. RvD2 activity likely involves regulation of innate immunological mechanisms at several regulatory checkpoints, which are necessary to prime pathogenic T-cell-mediated immunity.

## Ethics Statement

This study was carried out in accordance with the recommendations of the Guide for the Care and Use of Laboratory Animals (8th Edition), AAALAC. The protocol was approved by the Hebrew University Institutional Animal Care and Ethics Committee.

## Author Contributions

GM, TVD, and AW designed the study. GM and OH carried out the experiments. GM wrote the manuscript with support from TVD and AW.

## Conflict of Interest Statement

The authors declare that the research was conducted in the absence of any commercial or financial relationships that could be construed as a potential conflict of interest.
