# Supplementary figures and images for: Resolvin D2 Restrains Th1 Immunity and Prevents Alveolar Bone Loss in Murine Periodontitis
Source: Front Immunol. 2018 Apr 25;9:785. doi: 10.3389/fimmu.2018.00785 (PMC5996935; doi:10.3389/fimmu.2018.00785)

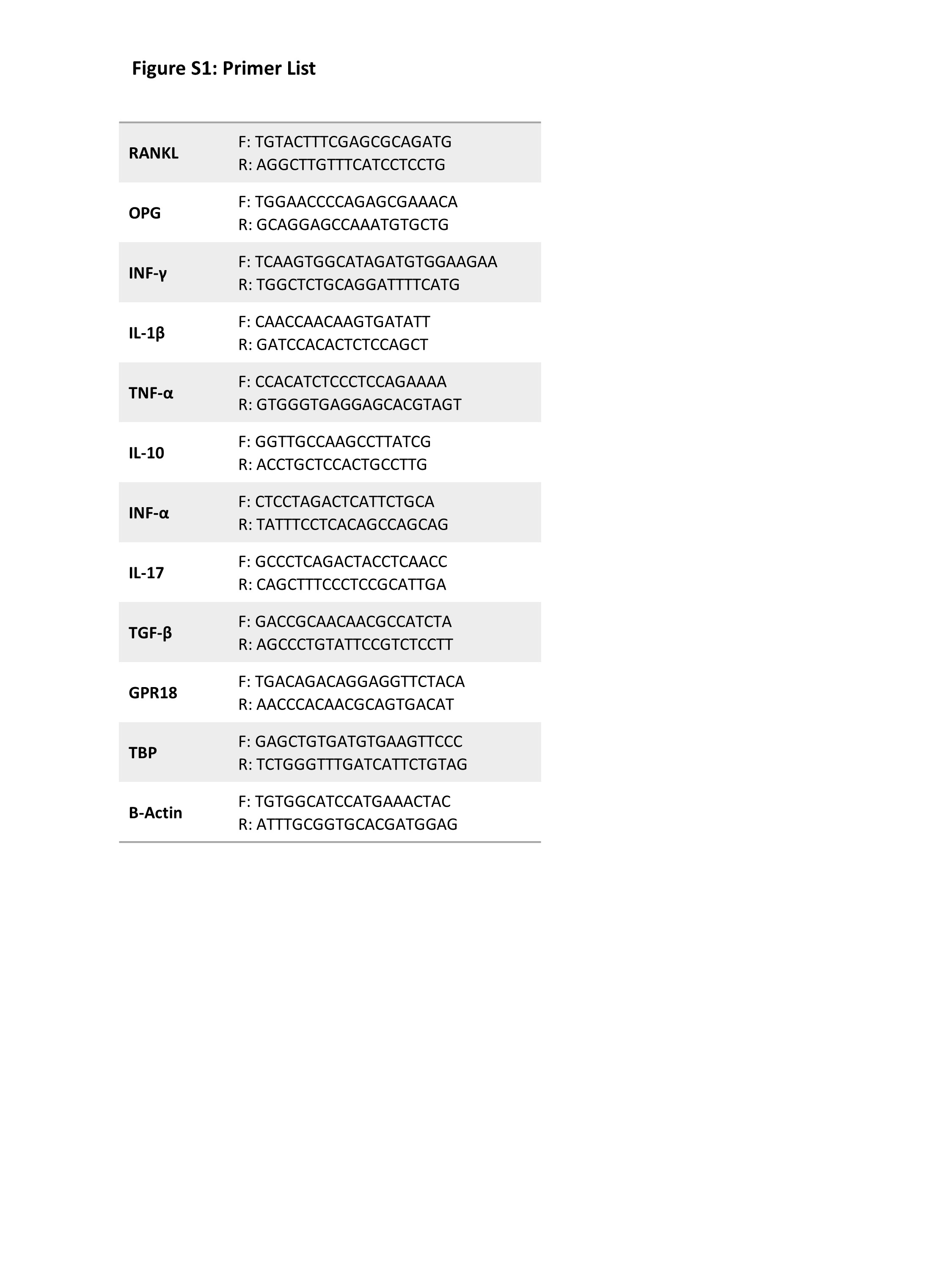

Supplement: Supplementary file 1 [file image_1.jpeg]

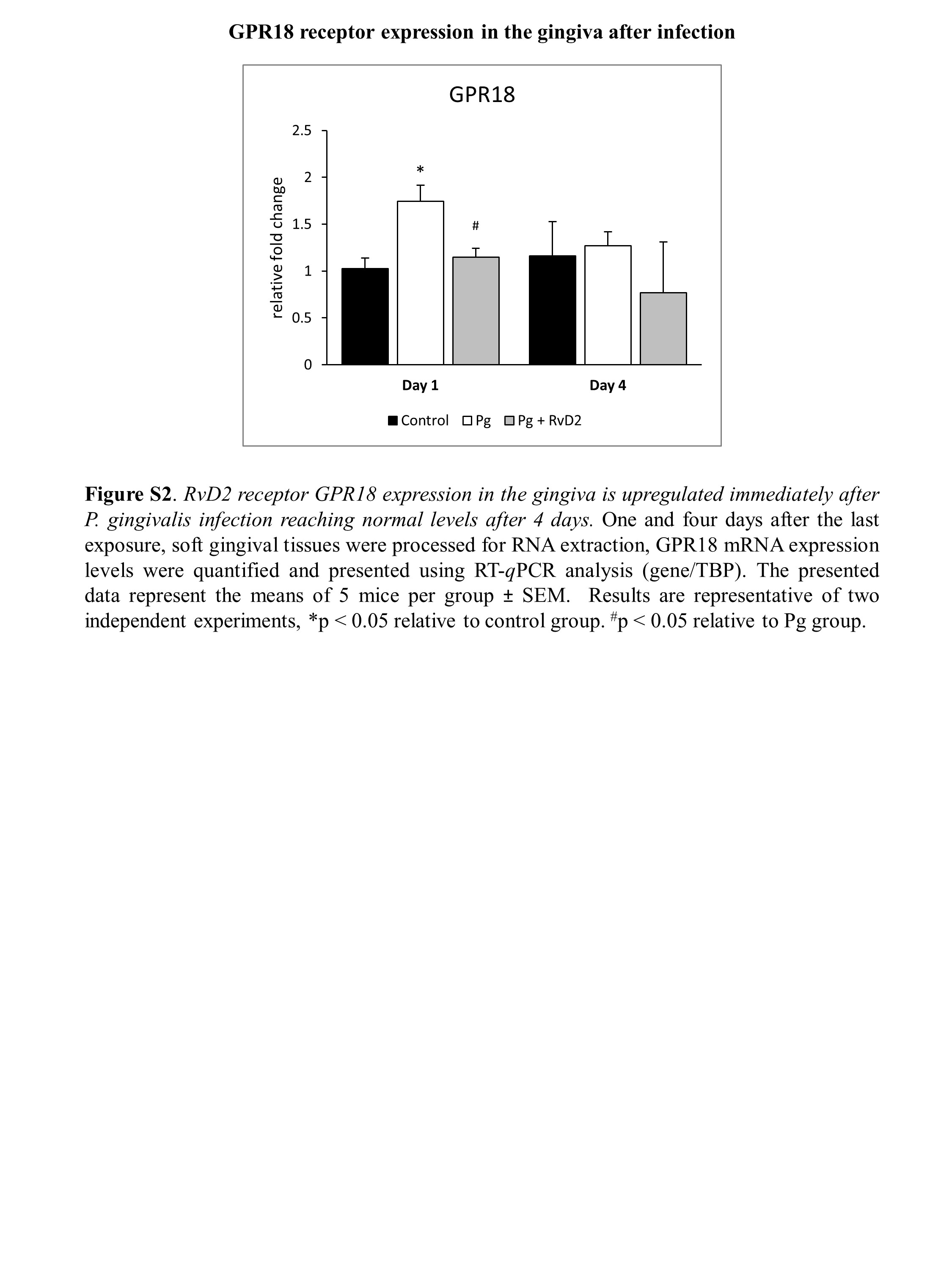

Supplement: Supplementary file 2 [file image_2.jpeg]
